# Supplementary material for: The interactome of intact mitochondria by cross-linking mass spectrometry provides evidence for coexisting respiratory supercomplexes
Source: Mol Cell Proteomics. 2017 Dec 8;17(2):216–32. doi: 10.1074/mcp.RA117.000470 (PMC5795388; doi:10.1074/mcp.RA117.000470)
Supplement: Supplemental Data [file supp_RA117.000470_133922_0_supp_23978_fzffwf.zip › spectra_annotation/mito_DR_spectra_annotation/309-1-7-1-10-1.pdf]

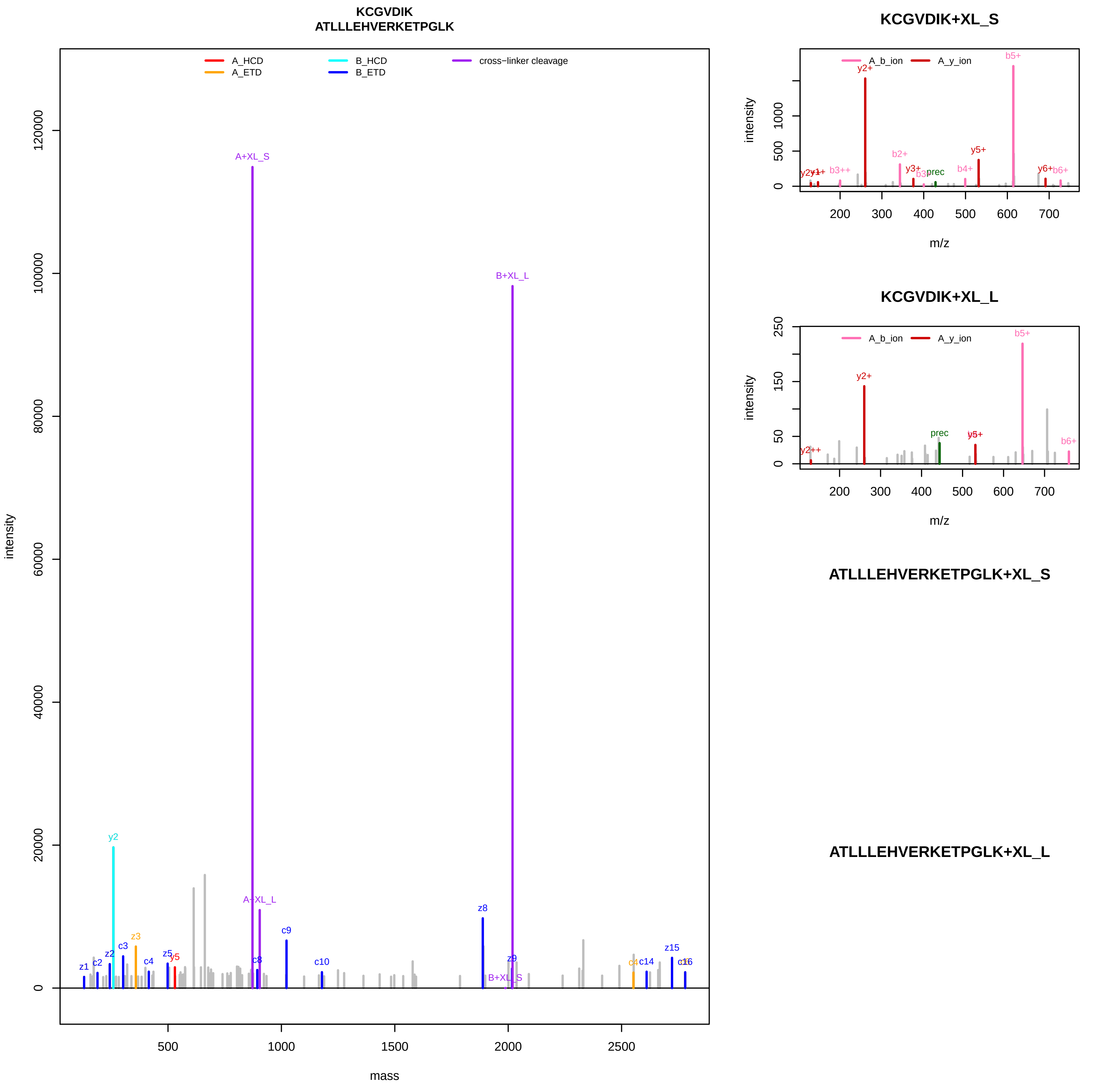
KCGVDIK
ATLLLEHVERKETPGLK
A_HCD
A_ETD
B_HCD
B_ETD
cross−linker cleavage
A+XL_S
B+XL_L
A+XL_L
B+XL_S
y2
z1
c2
z2
c3
z3
c4
z5
y5
c8
c9
c10
z8
z9
c4
c14
z15
c16
intensity
mass
0
20000
40000
60000
80000
100000
120000
500
1000
1500
2000
2500
KCGVDIK+XL_S
A_b_ion
A_y_ion
y2+
b5+
b2+
y3+
b3+
prec
b4+
y5+
y6+
b6+
y2+
y1+
b3++
intensity
m/z
0
500
1000
200
300
400
500
600
700
KCGVDIK+XL_L
A_b_ion
A_y_ion
y2+
b5+
prec
y5+
b6+
y2++
intensity
m/z
0
50
150
250
200
300
400
500
600
700
ATLLLEHVERKETPGLK+XL_S
ATLLLEHVERKETPGLK+XL_L
